# Determination of Bovine Lactoferrin in Powdered Infant Formula and Adult Nutritionals by Heparin Affinity Extraction and Reverse-Phase High-Performance Liquid Chromatography/Ultraviolet Detection (HPLC/UV): Single-Laboratory Validation, First Action 2021.10

**DOI:** 10.1093/jaoacint/qsae038

**Published:** 2024-05-04

**Authors:** Jennifer L Frueh, Peng Shu, Thomas R Vennard, Michael A Gray, Shay C Phillips

**Affiliations:** Reckitt/Mead Johnson Nutrition, 2400 West Lloyd Expressway, Evansville, IN 47721, United States; Reckitt/Mead Johnson Nutrition, 2400 West Lloyd Expressway, Evansville, IN 47721, United States; Seagen Inc, Bothell, WA, United States; Reckitt/Mead Johnson Nutrition, 2400 West Lloyd Expressway, Evansville, IN 47721, United States; Reckitt/Mead Johnson Nutrition, 2400 West Lloyd Expressway, Evansville, IN 47721, United States; Reckitt/Mead Johnson Nutrition, 2400 West Lloyd Expressway, Evansville, IN 47721, United States

## Abstract

**Background:**

Infant formulas, and pediatric and adult nutritional products, are being fortified with bovine lactoferrin (bLF) due to its beneficial impacts on immune development and gut health. Lactoferrin supplementation into these products requires an analytical method to accurately quantify the concentrations of bLF to meet global regulatory and quality standards.

**Objective:**

To develop and validate a lactoferrin method capable of meeting the AOAC INTERNATIONAL *Standard Method Performance Requirements* (SMPR^®^) 2020.005.

**Methods:**

Powder formula samples are extracted using warm dibasic phosphate buffer, pH 8, then centrifuged at 4°C to remove insoluble proteins, fat, and other solids. The soluble fraction is further purified on a HiTrap heparin solid-phase extraction (SPE) column to isolate bLF from interferences. Samples are filtered, then analyzed by LC–UV using a protein BEH C4 analytical column and quantitated using an external calibrant.

**Results:**

The LOQ (2 mg/100 g), repeatability (RSD: 2.0–4.8%), recovery (92.1–97.7%), and analytical range (4–193 mg/100 g) all meet the method requirements as stated in SMPR 2020.005 for lactoferrin.

**Conclusion:**

The reported single-laboratory validation (SLV) results demonstrate the ability of this lactoferrin method to meet or exceed the method performance requirements to measure soluble, intact, non-denatured bLF in infant and adult nutritional powder formulas.

**Highlights:**

The use of a heparin affinity column to isolate lactoferrin from bovine milk products combined with a selective analytical chromatographic column provides suitable analyte specificity without requiring proprietary equipment or reagents.

Lactoferrin is a human milk glycoprotein shown to have beneficial physiological effects, including modulating immune functions, protective activities against pathogens, and supporting intestinal health (1). Lactoferrin is also naturally present in bovine milk and some infant formulas, though innate concentrations are much lower than human milk. Since bLF has a reported bioactivity comparable to human lactoferrin (1, 2) and is commercially available as a purified ingredient, it could be used to fortify infant formula and adult nutritionals. For this reason, assays to detect and quantify bovine lactoferrin (bLF) have been developed utilizing an array of techniques, including ELISA (3, 4), UHPLC–MS/MS (5, 6), HPLC coupled with a heparin affinity column (7), and surface plasmon resonance (SPR) optical biosensor immunoassay (8, 9). The method described here has been optimized and validated to measure bLF in powdered infant formulas and adult nutritionals and is applicable to matrixes containing only lactoferrin of bovine origin. The previous version of this method was internally developed and validated at Reckitt/Mead Johnson Nutrition, Evansville, IN for milk-based infant formula matrixes. As new matrixes were introduced (e.g., partially hydrolyzed milk-based powder), modifications were made to maintain consistent bLF method performance and are summarized in Table 1.

**Table 1.** bLF method development modifications

Method parameter	Original method	Updated method
Standard curve	6.25–62.5 μg/mL	10–250 μg/mL
Initial sample hydration time^a^	20 min	1 h
LC column	Thermo Scientific BioBasic, C4 column, 5 μm, 4.6 × 250 mm	Waters Xbridge, C4 column, 3.5 μm,4.6 × 150 mm
LC flow rate	1.0 mL/min	0.5 mL/min

a *See* method section **F(a3)**.

## Single-Laboratory Validation

A single-laboratory validation (SLV) was conducted to validate this bLF method against the SMPR 2020.005 criteria listed in Table 2 (10). This SLV characterized the method performance for specificity, instrument response linearity, limits of detection and quantitation, repeatability, intermediate precision, and accuracy. Selectivity experiments to address heat-impacted (denatured) bLF and other milk proteins were also incorporated into the final round of SLV testing to completely evaluate SMPR 2020.005 guidelines.

**Table 2.** Method performance requirements for bLF (SMPR 2020.005)

Analytical range	4–200 mg/100 g
LOQ	4 mg/100 g
Recovery	90–110%
Repeatability (RSD_r_)	<6%
Reproducibility (RSD_R_)	<9%

Due to the limited commercial availability of infant formula and adult nutritional matrixes fortified with bLF, internally sourced materials were utilized to assess method performance. The validation samples are listed in Table 3. All sample results were analyzed as powder basis then converted to reconstituted values based on a reconstitution rate of 25 g powder + 200 g diluent (dilution factor = 9).

**Table 3.** Validation samples

Sample	Original submission samples Description
1	Milk-based infant formula powder
2	Milk-based partially hydrolyzed infant formula powder
3	Milk-based adult nutritional powder (bLF ≤14 mg/100g)
4	Milk-based infant formula powder (bLF ≤14 mg/100g)

Sample	Updated submission samples Description
5	Milk-based infant formula powder (bLF ≤14 mg/100g)
6	Milk-based toddler formula powder (bLF ≤14 mg/100g)
7	Milk-based adult nutritional powder (bLF ≤14 mg/100g)
8	Milk-based infant formula powder, overspiked based on ∼193 mg/100g bLF

### Specificity

- *Chromatographic integrity.*—Specificity was determined by comparing chromatograms from a solvent blank, a lactoferrin standard, and at least one matrix blank and sample per matrix family.
- *Heat-impacted lactoferrin.*—To assess the ability to differentiate intact, soluble lactoferrin from heat-impacted lactoferrin, a 10.0 mg/mL solution of lactoferrin standard (Cerilliant, L-047) was heated to 90°C for 60 min in duplicate on separate days. In each case, the heat-treated lactoferrin was spiked into a milk-based IF powder matrix blank at 56 mg/100g prior to sample preparation and analysis. These samples were compared to matrix blanks spiked at 56 mg/100g with unadulterated lactoferrin.
- *Other proteins.*—The molecular weights and isoelectric points for bovine milk proteins are calculated in Uniprot. These values show that bovine lactoperoxidase (LP, calculated average MW: 78.2 kDa, calculated isoelectric point (PI): 8.91) has similar physical properties to bLF (calculated average MW: 76.1 kDa, calculated PI: 8.67; 11, 12). The similarities suggest that lactoperoxidase may interfere with lactoferrin in the cleanup and chromatographic analysis. A solution of lactoperoxidase (Sigma-Aldrich, L2005) was analyzed both with and without the heparin column cleanup to assess the potential interference and was followed by a chromatographic evaluation.

Bovine lysozyme was also considered as a potential interference. The lack of a commercially available standard inhibited collecting any experimental data. However, its molecular weight and isoelectric point (MW ∼18 kDa, PI 9.5), and relatively low content in bovine milk, suggest it is even less likely to cause any substantial interference (13–15).

### Linearity

The linearity of the standard curve provides an assessment of the instrument response to the concentration of the analyte standards. The slope and intercept of the response define the standard curve and the correlation coefficient provides one assessment of the linearity. Six levels of standards were prepared and analyzed for slope, intercept, correlation, and percent deviation, to assess the accuracy of the calibration.

### LOD

The practical LOD was set at one-tenth the concentration of the lowest level calibration standard and was assessed by diluting the lowest standard (10 µg/mL) by 10 and performing six replicate injections of the resulting solution. The ratio of bLF signal to baseline noise was determined for each injection.

### LOQ

The LOQ for the analysis was set at the lowest standard level and assessed by performing six replicate injections of the lowest level standard. The ratio of bLF signal to baseline noise was determined for each injection.

### Repeatability and Intermediate Precision

Repeatability values were obtained from six replicate data points in a single run using six independent sample preparations for each sample, and the intermediate precision of the method was determined from single data points obtained on six different days.

### Method Equivalence Studies

To assess the impact of method modifications, equivalence studies were performed on two of the samples validated in the original SLV: milk-based infant formula powder and milk-based partially hydrolyzed infant formula powder. Since the matrixes had been fully validated using the same samples, the equivalency study was considered sufficient to demonstrate that the updated method produced equivalent results compared to the original version. Six Independent replicates for each matrix were prepared and analyzed using the method initially submitted to the Stakeholder Program on Infant Nutrition and Adult Nutritionals (SPIFAN), and six were prepared and anayzed using the updated method.

### Accuracy

Accuracy was tested by spiking each matrix with two levels of lactoferrin, roughly representing 50% and 100% overspike levels, using the certified reference standard as spiking agent. Recovery studies for each sample were analyzed in triplicate with individual recovery replicates performed on different days.

### Analytical Range

The required analytical range coverage is 4–200 mg/100g, based on reconstituted concentrations. The analytical range of the lactoferrin method is evaluated based on the standard curve range, as well as the demonstrated repeatability, intermediate precision, and accuracy results.

**AOAC *Official Method*^SM^ 2021.10**

**Bovine Lactoferrin in Powdered Infant Formula and Adult Nutritionals**

**Heparin Affinity Extraction and Reverse-Phase**

**High-Performance Liquid Chromatography/**

**Ultraviolet Detection (HPLC/UV)**

**First Action 2021**

[Applicable to the determination of soluble, intact, non-denatured bLF in bovine milk-based infant formula and pediatric/adult nutritional powders.]

*Caution:* Refer to the material safety data sheets of chemicals prior to use and follow the use of suggested personal protective equipment. Concentrated trifluoroacetic acid and o-phosphoric acid are corrosive and have strong pungent odors, and contact can cause severe burns. Work in the fume hood or in a well-ventilated area and wear safety gloves, lab coat, and protective eyeglasses. Acetonitrile and ethanol are highly flammable, and harmful if inhaled or in contact with skin. Wear protective clothing, gloves, and eyeglasses. Keep away from heat and open flame.

## A. Principle

Lactoferrin content is determined by reverse-phase (RP)-HPLC combined with a UV or a diode array detector (DAD) at 280 nm wavelength. Samples are dissolved in a sodium phosphate dibasic solution and then centrifuged to separate the liquid phase (whey) from fat and casein. bLF is then isolated and purified from the liquid phase using a HiTrap heparin affinity column as solid-phase extraction (SPE). Eluates from the heparin affinity column are then filtered and collected for HPLC analysis using a protein BEH C4 analytical column.

*Critical components.*—*(1)* A suitable bLF reference standard [*see* Note (2) in section **E(a)**] must be obtained and carefully weighed to avoid absorbed water weight. *(2)* The sample extract solution should be carefully handled to avoid any spills. *(3)* The HiTrap heparin column should not be allowed to run dry before the final bLF eluate collection.

## B. Apparatus

- *Liquid chromatograph.*—Waters Acquity I-Class UHPLC system (FTN module) with UV detector, or equivalent.
- *Analytical columns.—*Waters XBridge Protein BEH C4 column, 300 Å, 3.5 µm particle size, 4.6 × 150 mm, Part No. 186004504, or equivalent.
- *Analytical balance.*—Mettler Toledo with anti-static kit or equivalent, capable of weighing to ± 0.01 mg.
- *Centrifuge.*—Sorvall Biofuge Stratos, Model No. LR56495, or equivalent, with appropriate rotor, relative force to 8000*g* with cooling to 4°C.
- *pH meter.*—Thermo Scientific Orion 3 Star, or equivalent, with pH 4, 7, and 10 buffers accurate to ± 0.01.
- *Beakers.*—500 mL and 1000 mL.
- *Reagent bottle.*—100 mL.
- *Vortex mixer.*—VWR Analog Vortex Mixer, Model No. VM-3000, or equivalent.
- *Centrifuge tubes.*—Corning Falcon 15 mL and Conical centrifuge tubes, polypropylene 50 mL, Part Nos. 352096 and 352070, or equivalent.
- *Test tube rocker.*—Thermo Vari-Mix Test Tube Rocker, Model No. M48725, or equivalent.
- *Heparin HP affinity column.*—HiTrap Heparin HP affinity column, 1 mL, GE Healthcare, Code17-0406-01, or equivalent.
- *Syringe filters.*—Pall Life Sciences, 0.45 µm, wwPTFE Mini, 13 mm, Acrodisc, Part No. 2402, or equivalent.
- *Disposable syringe.*—BD Medical, 5 mL with Luer-Lok tips, Part No. 301027, or equivalent.
- *Disposable syringe.*—BD Medical, 10 mL with Luer-Lok tips, Part No. 301029, or equivalent.
- *Pipettors with tips.*—Eppendorf 10–100 µL, 100–1000 µL, 1–5 mL, 1–10 mL, or equivalent.
- *Transfer pipettes.*—Fisherbrand plastic disposable transfer pipettes stock, Part No. 13-711-9AM, or equivalent.
- *Autosampler vials.*—National Scientific 11 mm, 12 × 32, Clear Snap, silanized HPLC 2 mL vials, Part No. C4011S5W, or equivalent.
- *Silicone septa snap caps.*—Pre-slit, PTFE; National Scientific Stock, No. 03-396X, or equivalent.
- *Water bath.*—Buchi B-491, or equivalent.
- *Volumetric flasks.*—Kimax Kimble, class A: 5 mL, 10 mL, 25 mL, 100 mL, 500 mL, 1000 mL.
- *Vacuum manifold.*—Waters Vacuum Manifold Sep-Pak and/or Supelco Visiprep DL, or equivalent.
- *Adapters.*—Threaded Luer adapter (male Luer x female 10-32 threaded coned), Idex No. P-656 or P-676, or equivalent.
- *Adapters.*—Connector, 1/16” Male/Luer Female—Cytiva, Part No. 18-1112-51, or equivalent.
*Note*: One adapter is supplied in each box of five HiTrap columns.

## C. Reagents

- *Water*.—18.2 MΩ/cm, or equivalent.
- *Sodium hydroxide (NaOH).*—ACS grade, ≥98%, Fisher Scientific, Catalog No. RDC0530125, or equivalent.
- *Sodium phosphate dibasic (Na_2_HPO_4_).*—≥99.0%, Sigma-Aldrich, Stock No. 71640-250, or equivalent.
- *o-Phosphoric acid.*—ACS grade, ≥85%, Fisher Scientific, Catalog. No. A242-500, or equivalent.
- *Sodium chloride.*—Certified ACS grade, ≥99.3%, Fisher Scientific, Stock No. S271500, or equivalent.
- *Ethyl alcohol.*—200 proof (absolute), ≥99.5%, Sigma-Aldrich, Stock No. 459836, or equivalent.
- *Trifluoroacetic acid (TFA).*—≥99.9%, EMD Chemicals, Stock No. MTX1276-6, or equivalent.
- *Acetonitrile.*—HPLC grade or better, 99.9%, Fisher Chemical, or equivalent.
- *Lactoferrin (from bovine milk).*—Certified reference material, Cerilliant No. L-047, Supelco, or a standard that has been validated [*see* Note (2) in section **E(a)**].

## D. Reagent/Mobile Phase Preparation

*Note:* Reagent preparations may be scaled up or down as needed.

- *1 N Sodium hydroxide*.—Weigh 1.0 g NaOH into a 25 mL volumetric flask, add 20 mL water to dissolve and then bring to volume with water. Mix well before transferring it to a screw-type bottle for storage. Alternatively, 1 N NaOH solution can be purchased.
- *Diluted o-Phosphoric acid solution (0.425%)*.—Add 80 mL water to a 100 mL volumetric flask, carefully add 0.5 mL *o*-phosphoric acid into the flask, gently swirl and then bring to volume with water. This solution can be further diluted if needed.
- *0.2M Sodium phosphate dibasic buffer, pH 8.0 (loading buffer)*.—Weigh 28.4 g sodium phosphate dibasic into a 1000 mL beaker, add a stir bar and 900 mL water to dissolve, adjust pH to 8.0 ± 0.05 with *o*-phosphoric acid (or diluted) and 1 N sodium hydroxide solution, and then transfer the solution to a 1000 mL volumetric flask or to an equivalent container, dilute to a final volume of 1000 mL with water.
- *0.05 M Na_2_HPO_4_–1 M NaCl buffer, pH 8.0 (eluting buffer)*.—Weigh 3.55 g sodium phosphate dibasic and 29.2 g sodium chloride into a 500 ml beaker, add 450 mL water to dissolve, adjust pH to 8.0 ± 0.05 with *o*-phosphoric acid (or diluted) and 1 N sodium hydroxide solution, and then transfer the solution to a 500 mL volumetric flask or to an equivalent container, dilute to a final volume of 500 mL with water.
- *0.05 M Na_2_HPO_4_–2 M NaCl buffer, pH 8.0 (cleanup buffer)*.—Weigh 3.55 g sodium phosphate dibasic and 58.4 g sodium chloride into a 500 mL or larger beaker, add about 480 mL water to dissolve, adjust pH to 8.0 ± 0.05 with *o*-phosphoric acid (or diluted) and 1 N sodium hydroxide solution, and then transfer the solution to a 500 mL volumetric flask or to an equivalent container, dilute to final volume with water.
- *20% Ethyl alcohol*.—Combine 20 mL absolute ethyl alcohol with 80 mL water in a reagent bottle. Mix well.
- *Mobile phase A (0.1% trifluoroacetic acid solution).*—Add approximately 800 mL water to a 1000 mL volumetric flask, pipette 1 mL trifluoroacetic acid into the same flask and dilute to volume with water. Swirl to mix.
- *Mobile phase B (0.1% trifluoroacetic acid in acetonitrile solution)*.—Add approximately 800 mL acetonitrile to a 1000 mL volumetric flask, pipette 1 mL trifluoroacetic acid into the same flask and dilute to volume with acetonitrile. Swirl to mix.

## E. Preparations of Standard Solutions

*Note:* Standard preparations may be scaled up or down as needed.

- *bLF stock solution I (10 mg/mL).*—Accurately weigh 90–120 mg bLF reference material into a 10 mL volumetric flask. Record the weight to 0.01 mg. Add 5–8 mL water and agitate gently to mix. Allow the solution to stand at room temperature for at least 1 h until dissolved. Dilute to volume and gently mix. Store at 4°C in a sealed plastic vessel protected from light. Calculate the concentration (conc) to four significant figures as follows:
  $$\;\mathrm{Conc}\;\left(\mathrm{bLFStock}\;I\right)=\frac{\mathrm{Mass}\;\left(\mathrm{Cert}.\;\mathrm{Ref}.\;\mathrm{Std}\right)\;\times \;\frac{\mathrm{Purity}\;\left(wt.\;\%\right)}{100}}{\mathrm{Vol}\;\left(\mathrm{Water}\right)}$$
  where, Conc (bLFStock I) = concentration of bLF stock solution I; Mass (Cert. Ref. Std) = mass of the certified reference standard; Vol (Water) = the volume of water added to the certified reference standard (10.0 mL).
  Alternatively, accurately weigh 80–120 mg bLF reference standard material into a 15 mL centrifuge tube. Record the weight to 0.01 mg. Calculate the net bLF mass by multiplying the reference standard weight by the purity (wt. %). Calculate and add the volume of water required to reach a concentration of 10.00 mg bLF/mL to the tube. Gently swirl every 10–15 min to dissolve and let the solution stand for at least 1 h at room temperature. Store at 4°C in a sealed plastic vessel protected from light. Calculate the concentration to four significant figures as follows:
  $$\begin{array}{c} M\mathrm{ass}\;\left(\mathrm{Cert}.\;\mathrm{Ref}.\;\mathrm{Std}\right)\;\times \;\frac{\mathrm{Purity}\;\left(wt.\;\%\right)}{100}\;=\;\mathrm{Net}\;\mathrm{mass}\;(\mathrm{Cert}.\mathrm{Ref}.\mathrm{Std})\\ \begin{array}{c} \frac{\mathrm{Net}\;\mathrm{mass}\;(\mathrm{Cert}.\mathrm{Ref}.\mathrm{Std})}{10.00\;mg/mL}\;=\;\mathrm{Vol}\;\left(\mathrm{Water}\right)\\ \frac{\mathrm{Net}\;\mathrm{mass}\;(\mathrm{Cert}.\mathrm{Ref}.\mathrm{Std})}{\mathrm{Vol}\;\left(\mathrm{Water}\right)}=\mathrm{Conc}\;\left(\mathrm{bLF}\;\mathrm{Stock}\;I\right) \end{array} \end{array}$$
  Notes on bLF stock solution I:
*(1)* bLF reference materials may be hygroscopic. Please ensure the reference standard is at room temperature before opening and work quickly when weighing out.
*(2)* Lactoferrin standards may be made from either certified reference standard material or from lactoferrin material that has been validated against a certified reference standard (assessed in at least triplicate over a minimum of 2 days by LC using a certified reference standard for external calibration and stored sealed in a desiccator to prevent degradation over the shelf life of the standard).
- *bLF stock solution II (∼0.5 mg/mL).*—Pipette 1.25 mL bLF stock solution I and dilute to 25 mL with 0.05 M Na_2_HPO_4_–1 M NaCl buffer, pH 8.0 (eluting buffer) in a volumetric flask. Store at 4°C in a sealed plastic vessel. Calculate the concentration to four significant figures as follows:
  $$\mathrm{Conc}\;\left(\mathrm{bLF}\;\mathrm{stock}\;II\right)=\mathrm{Conc}\;\left(\mathrm{bLF}\;\mathrm{stock}\;I\right)\;\times \;V(\mathrm{bLF}\;\mathrm{stock}\;I)/V(\mathrm{bLF}\;\mathrm{stock}\;II)$$
  where, Conc (bLF stock II) = the concentration of bLF stock solution II; conc (bLF stock I) = the concentration of bLF stock solution I; V(bLF stock I) = the volume of bLF stock solution I used (1.25 mL); V(bLF stock II) = the total volume of bLF stock solution II made.
- *bLF working standards.*—Pipette the specified volumes of bLF stock solution II and 0.05 M Na_2_HPO_4_–1 M NaCl buffer, pH 8.0 (eluting buffer) into centrifuge tubes as indicated in Table **2021.10A** for each calibration standard.Alternatively, solutions may be made up in 10.0 mL volumetric flasks. Mix well and filter into a silanized HPLC vial through a 0.45 µm, 13 mm wwPTFE Mini syringe filter for analysis. Prepare fresh daily. Calculate the concentrations to four significant figures as follows:
  $$\;\mathrm{Conc}\;\left(WS\right)=\mathrm{Conc}\;\left(\mathrm{StkII}\right)\;\times \;V(\mathrm{bLF}\;\mathrm{stock}\;II)/V(WS)$$
  where, Conc (WS) = the concentration of the working standard; Conc (bLF stock II) = the concentration of the bLF stock solution II; V (bLF stock II) = the volume of the bLF stock solution II used to make each working standard; V (WS) = the final volume of the working standard.

**Table 2021.10A.** Preparation of bLF working standard solutions

Calibration points	1	2	3	4	5	6
Nominal concentration, µg/mL	10	20	50	100	150	250
Volume (bLF stock solution II), mL	0.200	0.400	1.00	2.00	3.00	5.00
Volume (0.05 M Na_2_HPO_4_–1 M NaCl buffer, pH 8.0), mL	9.80	9.60	9.00	8.00	7.00	5.00

## F. Sample Preparation

- *Sample hydration*
  1. Accurately weigh 0.5–1.50 g sample, or enough to contain 0.5–10 mg lactoferrin, directly into a 15 mL centrifuge tube. Record the sample weight to 0.0001g.
  2. Pipette 11.5 mL warm (40°C) 0.2 M sodium phosphate dibasic solution, pH 8.0 (loading buffer) to the centrifuge tube in step *(1)*, vortex and/or shake well until smooth.
  3. Continue mixing the solution in step *(2)* by test tube rocker or shaker for at least 60 min, check the solution during the rocking, vortex mixing or shaking manually if needed.
  4. Centrifuge for at least 20 min at 8000*g* at 4°C.
  5. Carefully transfer as much of the liquid layer between the fat (top) and solid (bottom) layers in the centrifuge tube as is practicable, while minimizing disturbance of other layers, to a 25 mL volumetric flask.
  6. Pipette 11 mL warm (40°C) 0.2 M sodium phosphate dibasic solution, pH 8.0 (loading buffer) into the same centrifuge tube in step *(1)* and use a vortex mixer to mix the contents for 10 s or until fully suspended. Continue mixing by rocking for at least 20 min on the test tube rocker or shaker.
  7. Centrifuge the solution for 20 min at 8000*g* at 4°C and then repeat step *(5)*, using the same transfer pipette (if applicable) from step *(5)* to transfer the second liquid layer to the same 25 mL volumetric flask.
  8. Dilute the solution to volume (25 mL) with 0.2 M sodium phosphate dibasic solution, pH 8.0 (loading buffer).
  9. Thoroughly mix the combined solution in the volumetric flask.
- *Prepare the HiTrap heparin column [see section **F(d)** for full details on the use of the HiTrap columns]*
  1. Connect a 10 mL syringe barrel to the heparin affinity column using a syringe fitted to the Luer connector.
  2. Remove the snap-off end (for a new HiTrap heparin column) or unscrew the cap (for used) at the column outlet. Tightly connect the column outlet to a vacuum manifold with a threaded Luer adapter.
  3. Equilibrate the heparin affinity chromatography column by passing 5 mL 0.2 M sodium phosphate dibasic solution, pH 8.0 (loading buffer) through the column and discard the eluent. Notes about column usage can be found in section **F(d)**.
- *Sample purification*
  1. Ensure the extract solution from step **F(a9)** above is mixed well and then pipette 5 mL extract solution into the syringe barrel. Drain the solution through the column with vacuum and discard the eluent. *Note*: Ensure the total amount of lactoferrin being loaded onto the column is at least 0.1 mg.
  2. Wash the column, using vacuum, with 10.0 mL 0.2 M sodium phosphate dibasic solution, pH 8.0 (loading buffer) and discard the eluent.
  3. Remove the column assembly from the vacuum manifold. Elute the column with 4.50 mL 0.05 M Na_2_HPO_4_–1 M NaCl solution, pH 8.0 (eluting buffer), collecting the eluent into a 5.0 (or 10.0 mL) volumetric flask or equivalent container.
  4. Dilute to volume with 0.05 M Na_2_HPO_4_–1 M NaCl solution, pH 8.0 (eluting buffer) and mix well. *Note*: The final volume (size of volumetric flask for collecting eluent) can be adjusted so that the bLF level falls on the standard curve.
  5. Filter a portion of mixed solution through a 0.45 µm, 13 mm wwPTFE Mini syringe filter into a silanized HPLC vial for analysis.
  6. After collecting the final bLF eluent from the purification process, wash the column using the vacuum manifold with 5 mL of 0.05 M Na_2_HPO_4_–2 M NaCl solution, pH 8.0 (cleanup buffer) and then 5 mL 20% ethanol. Discard eluents after washing and store the column in 20% ethanol at RT for reuse up to four times. *Note*: Discard column if sample matrix binds on column during purification step, indicated by significant restriction in flow.
- *Care and use of HiTrap heparin columns as SPE*
  1. Each column may be used up to four times. However, if there is difficulty loading, washing, and eluting a sample, do not use the column again.
  2. Columns are connected to the vacuum manifold using a threaded Luer adapter. Syringe barrels are connected to the tops of columns with a 1/16” male/Luer female connecter. One top connecter is included in each box of five columns. When suing a column for the first time, a plastic cap must be snapped off the bottom of the column.
  3. Equilibrate the columns with 5 mL loading buffer. After loading, use 10 mL loading buffer to wash away interferences. Discard the eluent from equilibrating, loading and wash steps.
  4. The recommended flow rate through the HiTrap heparin column is 0.1 to 1 mL/min for the 1 mL columns.
  5. Do not let the heparin column run dry during loading and washing of the sample. If you do allow the loading buffer to run out, you can add another couple of milliliters of loading buffer and allow it to drain to the bottom of the column.
  6. During loading, washing and eluting steps, the flow through the column may need to be assisted with the use of the plunger.
  7. The minimum load for adequate recovery on these specific columns in this size (1 mL) is 100 µg.
  8. When eluting lactoferrin, the column is removed from the vacuum manifold and eluent is collected into an appropriately sized volumetric flask.
  9. Columns are to be stored in storage solution (20% ethanol) at room temperature (RT).

## G. HPLC Instrument Parameters

- *Instruments.*—A Waters Acquity I-Class UHPLC system with flow-through needle and UV detector or equivalent. (The HPLC system should be located in an area where temperature fluctuations will be minimal throughout the run.)
- *Column.*—A Waters Bridge Protein BEH C4 column, 4.6 × 150 mm. Part No. 186004504 or equivalent.
- *Instrument settings.*—*See*  Table **2021.10B**.
- *Gradient.*—*See*  Table **2021.10C**.
- Turn on the detector and pump mobile phase over the column at a flow rate of 0.5 mL/min with the initial gradient conditions from Table **2021.10C** for at least 30 min to equilibrate the system.
- *Injection sequence:*—*See*  Table **2021.10D**.
*Note*: The sample stability at RT has not been evaluated past 24 h.
- *End of run.*—After all samples and standards have been analyzed, it is recommended to inject water three times: twice to clean out the UHPLC system and once to bring the instrument to standby mode.

**Table 2021.10B.** Instrument settings

Column temperature, ^o^C	35 ± 2
Sample tray temperature, ^o^C	Room temperature
Wavelength, nm	280
Flow rate, mL/min	0.5
Injection volume, µL	50
Run time, min	16

**Table 2021.10C.** HPLC gradient conditions

*Note*: Allow the system to equilibrate at tde initial gradient conditions for 30 min prior to beginning the analysis.			

Typical LC time program^a^	Time, min	% A	% B
	0	70	30
	5	55	45
	10	40	60
	12	30	70
	14	70	30

a Changes in the chromatographic conditions may be made to optimize chromatography on different chromatographic systems.

**Table 2021.10D.** Injection sequence^a^

Sample	No. of injections	Type
Water	1	Blank
Reagent blank	1	Sample
Middle bracketing standard	6	Control
Reagent blank	1	Sample
Calibration standards (1–6)	1 each	Standard
Reagent blank	1	Sample
Middle bracketing standard	1	Control
Check sample (if used)	1	Sample
Samples 1–9	1 each	Sample
Middle bracketing standard	1	Control
Samples 10–19	1 each	Sample
Middle bracketing standard	1	Control
Samples 20–29	1 each	Sample
Middle bracketing standard	1	Control
Water	2	Blank
Water	1	Blank (standby method)

a In the injection sequence, an extra blank of water or 0.05 M Na_2_HPO_4_–1M NaCl solution can be added after an anticipated higher bLF sample injection to avoid carryover.

## H. System Suitability/Analytical QC

- *Calibration curve.*—Every sequence must include a set of calibration standards covering the analytical range. The coefficient of determination (*r*^2^) of the standard curve must be a minimum of 0.995. The measured concentration of each standard level must be within ±10% of the theoretical value. A 1/x weighting may be used if an excessive bias is apparent in the low standards.
- *Check standards*.—Equilibration injections, followed by a set of six replicate injections of the check standard (middle standard) must be made prior to the calibration curve. The RSD, %, of the bLF peak areas for the six check standard injections must be ≤3.0%. The check standard must also be injected throughout the analytical set for bracketing, with the initial bracket standard run after the calibration standards and no more than 10 injections between bracketing check standards. The overall RSD of all the check standard peak areas must be ≤5%. Retention times of bracketing standards must be within ±5% agreement with the average retention time of the six replicate check standard injections. The retention time of the sample peaks must fall within ±10% agreement with the average check standard retention time. If any bracketing standard fails in the middle of a sequence, the data beyond the last successful check standard is to be considered invalid.

## I. Quantification

- Plot a linear external calibration curve by using the standard concentration values in units of µg/mL versus the integrated area of each bLF peak. Display the regression trendline equation:
  y=MC+b
  where *C* = concentration of bLF in the sample, (µg/mL); y = area of the bLF peak in the injected standard solution; b = y-intercept of the curve; and M = slope of the curve.
- Display the *R*^2^ value for the curve. The R^2^ value must be ≥0.995.
- Calculate the bLF concentration (mg bLF/100 g) in the sample using the following equation:
  $$\;\mathrm{bLF}\;\mathrm{Concentration}\;\left(\frac{mg}{100g}\right)=\;\frac{C}{m}\;\times \;V1\;\times \;\frac{V3}{V2}\;\times \;\frac{100\;(\mathrm{per}\;100g)}{1000\;\left(\frac{µg}{mg}\right)}\;$$

where *C* = bLF concentration derived from the standard calibration curve (µg/mL); *m* = sample weight (g); *V1 *=* *sample volume after centrifuge steps, combining both liquid layers and diluting to volume (25 mL); *V2 = *volume loaded onto the HiTrap column (5 mL); and *V3 = *final collection volume after HiTrap column (5 or 10 mL)

## Results and Discussion

### Specificity

**Figure 1.**
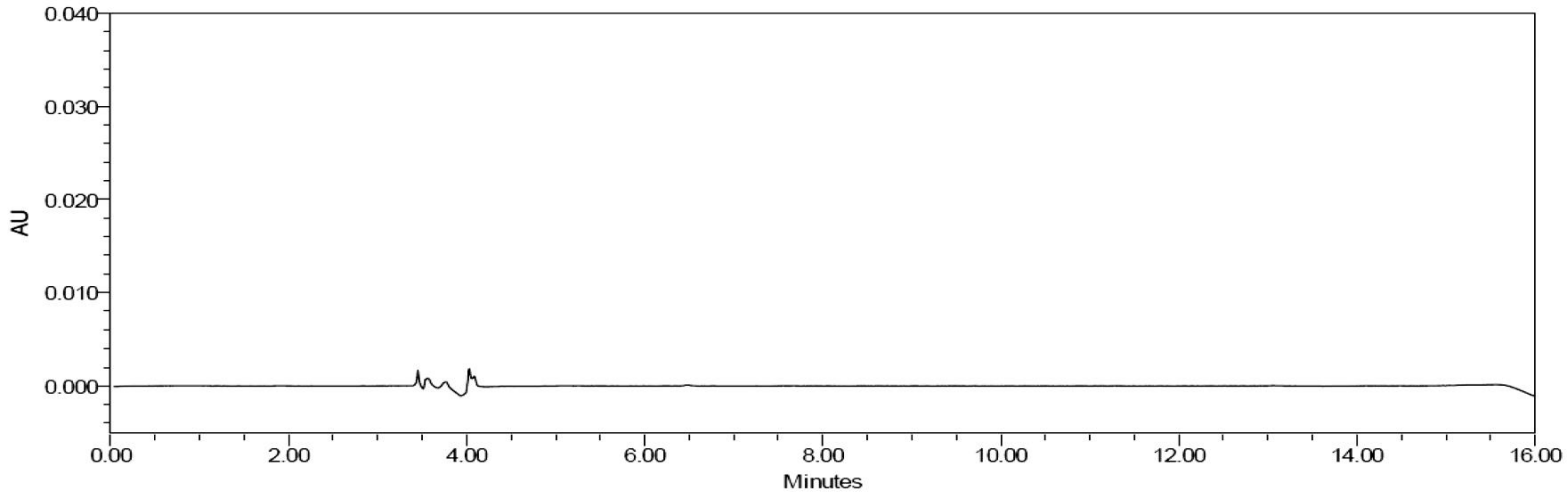
Solvent blank (unfortified).

**Figure 2.**
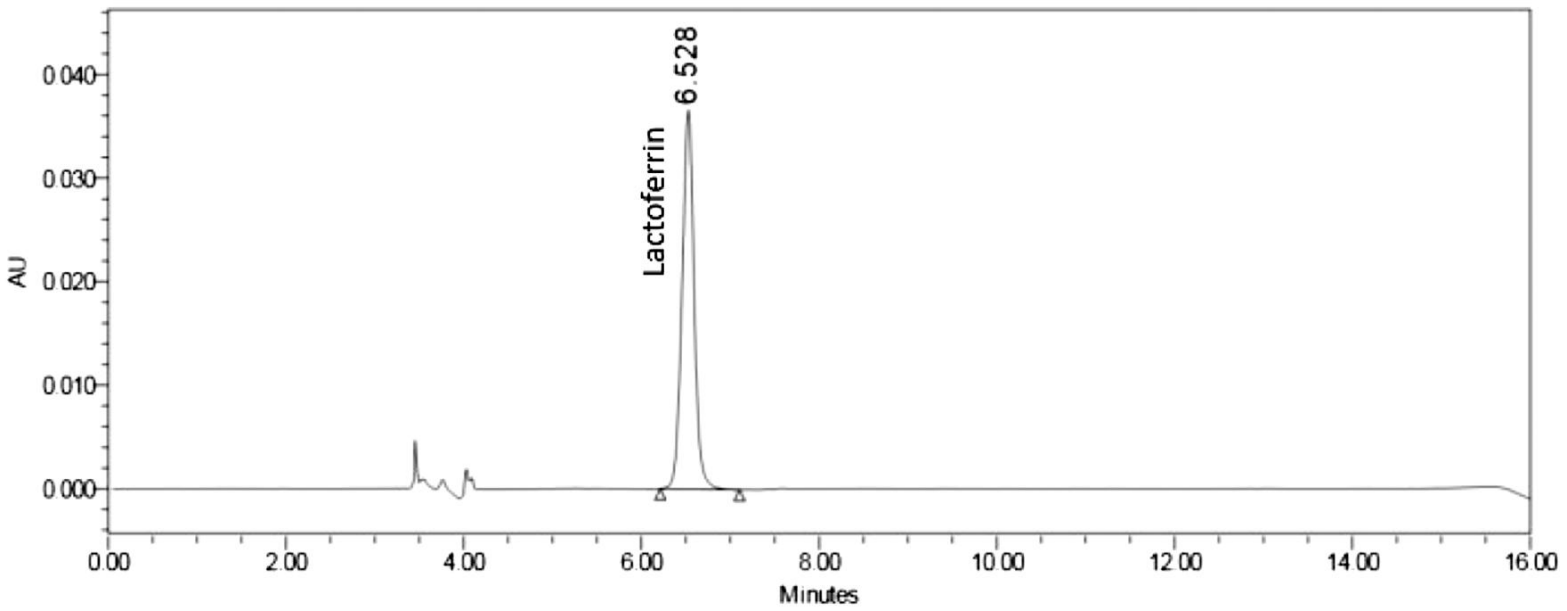
bLF standard (47.7 µg/mL).

**Figure 3.**
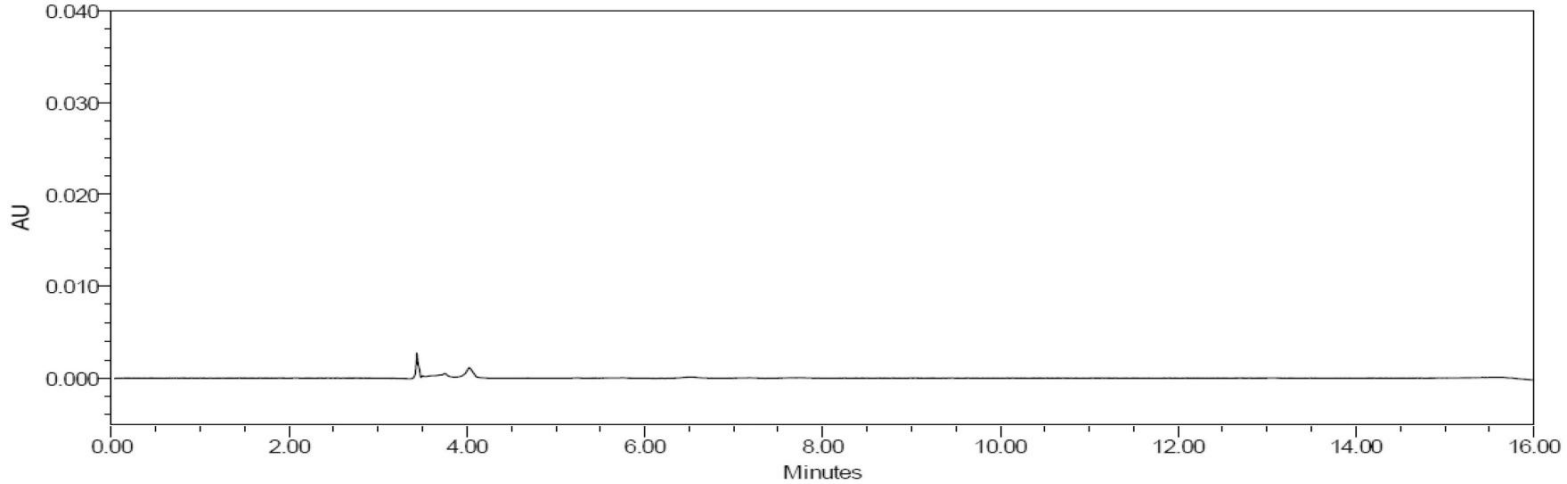
Sample—milk-based infant formula powder (matrix blank, unfortified).

**Figure 4.**
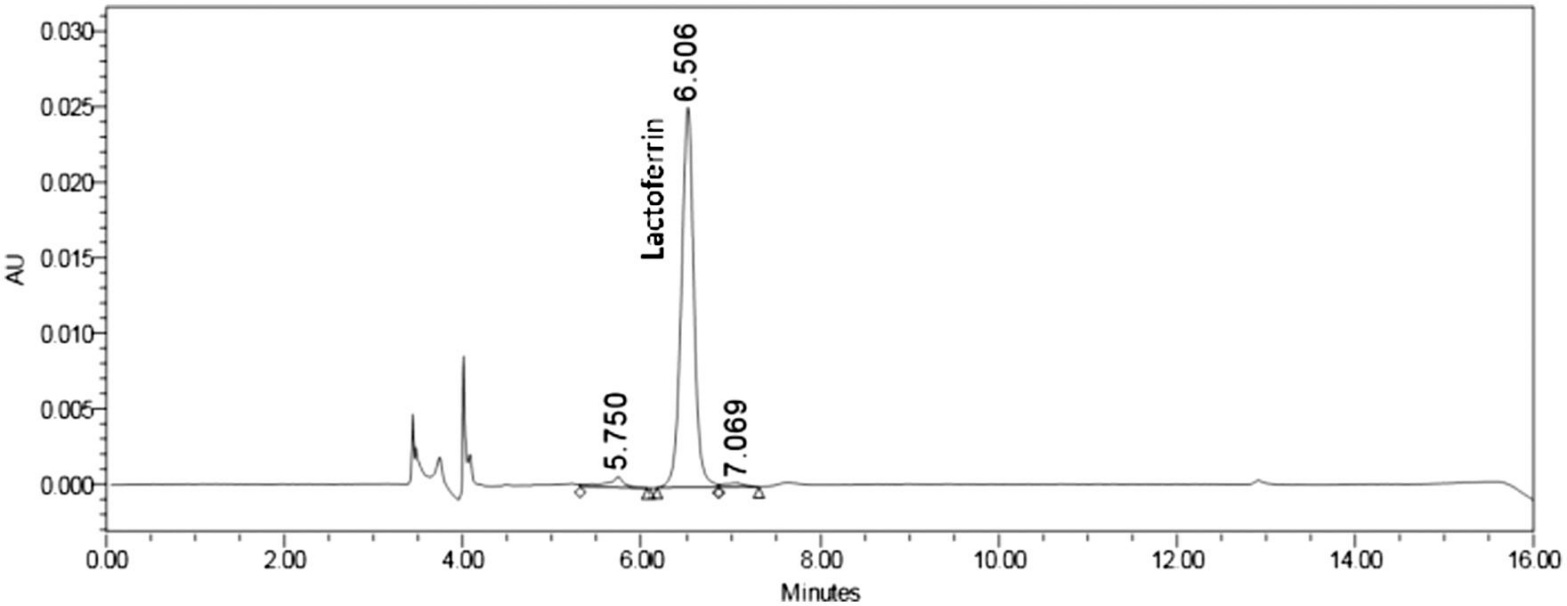
Sample—milk-based infant formula powder (bLF≤14 mg/100g).

**Figure 5.**
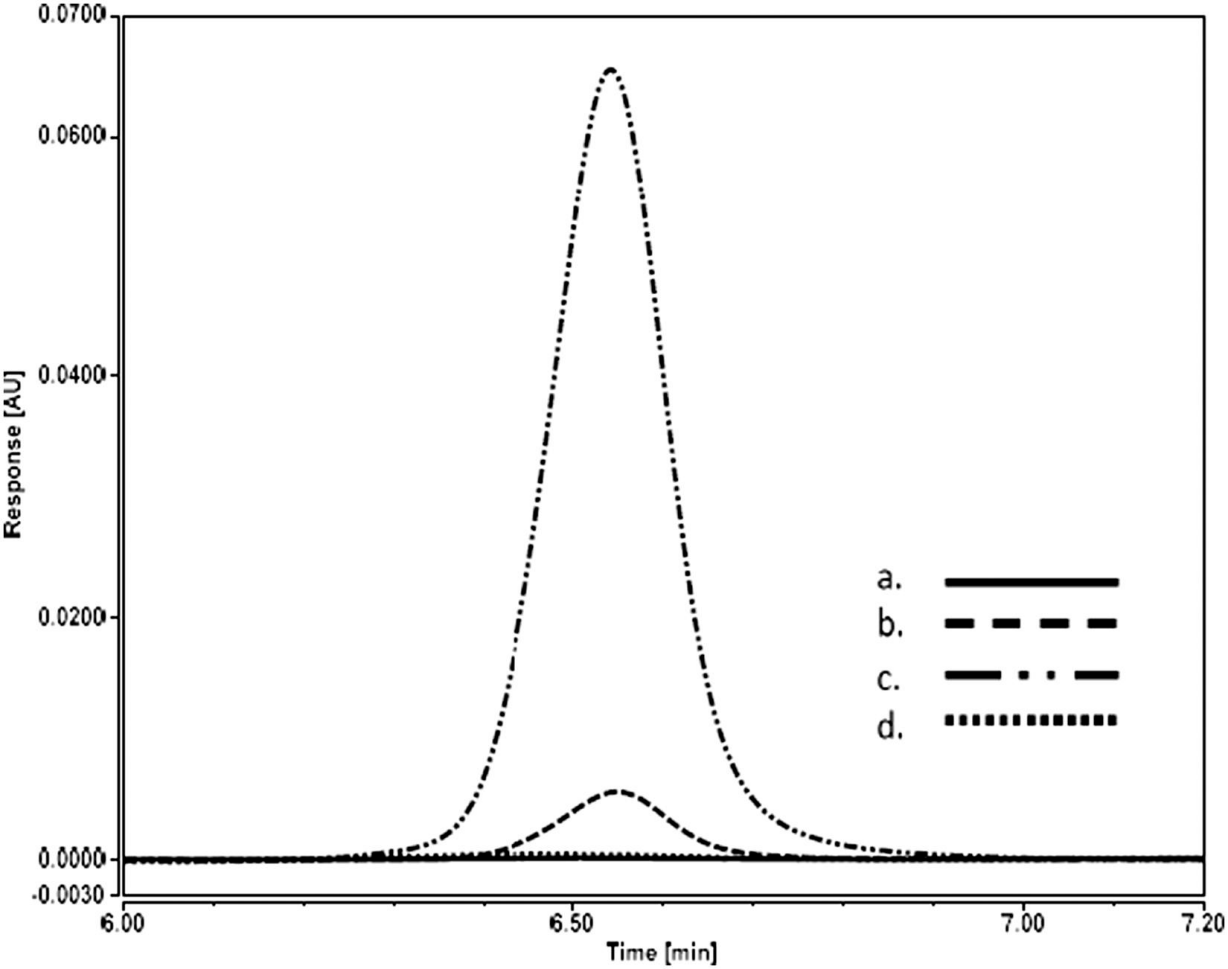
Overlay: (a) matrix blank, (b) bLF standard (10 µg/mL), (c) matrix blank + bLF (56 mg/100g), (d) matrix blank + heat-treated bLF (56 mg/100g).

**Figure 6.**
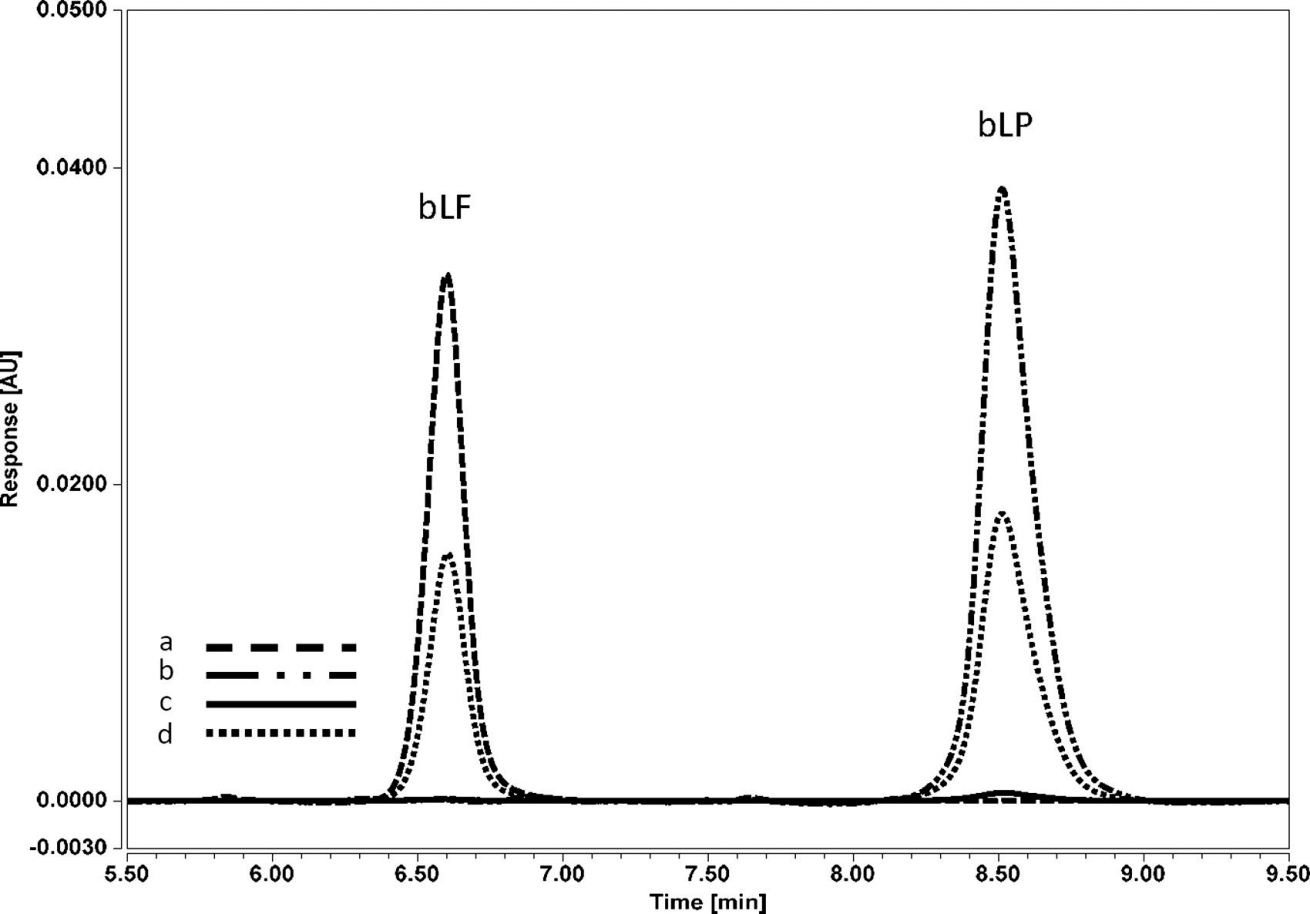
Overlay: (a) bLF standard (50 µg/mL), (b) lactoperoxidase (90 µg/mL, no heparin column), (c) lactoperoxidase, (90 µg/mL, with heparin column), (d) mix of lactoferrin (25 µg/mL) and lactoperoxidase (45 µg/mL, no heparin column).

- *Chromatographic integrity.*—Example chromatograms for the unfortified solvent blank, bLF standard, milk-based infant formula powder matrix blank, and milk-based infant formula powder (bLF ≤14 mg/100g) are shown in Figures 1–4. Lactoferrin was not detected in the blank or unfortified samples. Interference peaks were not identified in any of the injections. bLF retention times of samples were within ±10% of the standard.
- *Heat-impacted lactoferrin.*—All results of heat-treated lactoferrin spiked into matrix blanks were <LOQ, demonstrating that the method measures only soluble, intact, non-denatured lactoferrin. An overlay of chromatograms including a matrix blank, the lowest level lactoferrin standard (LOQ), the matrix blank + lactoferrin, and the matrix blank + heat-treated lactoferrin is shown in Figure 5, demonstrating that heat-impacted bLF was not detected using this method.
- *Lactoperoxidase.*—A solution of lactoperoxidase (Sigma-Aldrich, L2005) was analyzed both with and without the heparin column cleanup to assess the potential interference, and was followed by a chromatographic evaluation of a mixed lactoferrin/lactoperoxidase sample solution. An overlay of these experiments is shown in Figure 6, demonstrating that not only does the heparin column eliminate nearly all the lactoperoxidase, but the chromatographic column is also sufficiently selective to resolve the two components.

### Linearity

Linearity data are based on a standard curve of 10 µg/mL to 250 µg/mL, corresponding to an analytical range of 2 mg/100g to 278 mg/100g. Six levels of standards were prepared and analyzed on three standard runs on three days and data are summarized in Table 4.

**Table 4.** Linearity summary with bLF standard concentrations 10–250 µg/mL

	Day 1	Day 2	Day 3
Slope	7.55e3	7.58e3	7.44e3
Intercept	−1.15e4	−1.63e4	−8.96e3
Correlation coefficient	1.0000	1.0000	0.9999

**Standard conc, µg/mL**	**% Deviation**	**% Deviation**	**% Deviation**

9.544	4.5	4.6	−4.8
19.09	−1.4	−0.1	11.5
47.72	−0.5	−0.5	−2.1
95.44	−0.3	−0.5	−1.1
143.2	0.3	0.1	−0.3
238.6	−0.1	0.0	0.3

Average deviation, %	0.4	0.6	0.6

### LOD

The practical LOD was made by diluting the lowest calibration standard (10 µg/mL) by 10, yielding a standard solution corresponding to 0.2 mg/100g. Six replicate injections of this solution were made, the ratio of bLF signal to baseline noise for each injection was determined, and the results are displayed in Table 5. All individual S/N values were 8 or higher.

**Table 5.** LOD assessment with bLF standard concentration at 1 µg/mL (corresponds to 0.2 mg/100g in samples)

Replicate	S/N
1	8.54
2	8.20
3	8.47
4	10.49
5	9.13
6	9.96

Average	9.13
Acceptance criteria	S/N ≥3

### LOQ

The LOQ for the analysis is set at the lowest standard level of 10 µg/mL and determined through back calculation using the default weights and dilution factors to be 2 mg/100 g, reconstituted. The LOQ required by the SMPR for lactoferrin is 4 mg/100g, reconstituted. The peak area and S/N results of six replicate injections of the lowest standard from one analytical run are displayed in Table 6.

**Table 6.** LOQ assessment with bLF standard concentration at 9.54 µg/mL (corresponds to 2 mg/100g in samples)

Replicate	9.54 µg/mL peak area	S/N
1	64452	180.1
2	64575	202.6
3	64847	202.8
4	64529	234.2
5	64390	184.4
6	64381	247.9

Average	64529	208.7
Acceptance criteria		S/N ≥10

### Repeatability (RSD_r_) and Intermediate Precision (RSD_Int_)

The repeatability RSD_r_ was obtained for seven different lactoferrin fortified, milk-based powder samples and is summarized in Tables 7 and 8. Both RSD_r_ and RSD_Int_ for all matrixes studied fall below the SMPR RSD_r_ acceptance criteria.

**Table 7.** bLF repeatability (RSD_r_) and intermediate precision (RSD_Int_) summary (*n* = 6 each), original method validation

Sample	Description	Repeatability		Intermediate precision	
		Mean, mg/100g	RSD_r_, %	Mean, mg/100g	RSD_Int_, %
1	Milk-based infant formula powder	48.1	2.2	51.2	2.7
2	Milk-based partially hydrolyzed infant formula powder	40.0	3.2	41.0	3.4
3	Milk-based adult nutritional powder (bLF ≤14 mg/100g)	10.3	2.0	10.5	4.8
4	Milk-based infant formula powder (bLF≤ 14 mg/100g)	5.1	1.8	5.1	2.0

		Overall:	2.3		3.2
	Acceptance criteria:		<6%		<6%

**Table 8.** bLF repeatability (RSD_r_) and intermediate precision (RSD_Int_) summary (*n* = 6 each), updated method validation

Sample	Description	Repeatability		Intermediate precision	
		Mean, mg/100g	RSD_r_, %	Mean, mg/100g	RSD_Int_, %
5	Milk-based infant formula powder (bLF ≤14 mg/100g)	7.0	1.7	7.3	2.3
6	Milk-based toddler formula powder (bLF ≤14 mg/100g)	3.8	3.9	4.1	4.2
7	Milk-based adult nutritional powder (bLF ≤14 mg/100g)	5.8	4.7	5.8	2.7

		Overall:	3.4		3.1
	Acceptance criteria:		<6%		<6%

### Method Equivalence Studies

Equivalency studies were performed on two of the samples validated in the original SLV: milk-based infant formula powder and milk-based partially hydrolyzed infant formula powder. A summary of the results, demonstrating good agreement between the two methods, is shown in Table 9.

**Table 9.** bLF method equivalency summary (*n* = 6 each)

		Results		Results		
		Original method		Updated method		
Sample	Description	Mean, mg/100g	RSD, %	Mean, mg/100g	RSD, %	% Difference
1	Milk-based infant formula powder	42.3	1.8	42.3	2.6	0.0%
2	Milk-based partially hydrolyzed infant formula powder	35.0	1.6	34.9	2.8	0.3%

		Acceptance criteria:	<6%		<6%	≤8%

### Accuracy

The accuracy results as determined by mean recoveries for the original method are summarized in Table 10, and in Table 11 for the updated method. Included is a matrix blank spiked at high bLF levels (97 mg/100g and 193 mg/100g) to assess recovery at the upper end of the SMPR analytical range. All average recoveries for each sample matrix fell within the accepted range of 90–110%.

**Table 10.** bLF recovery analysis summary (*n* = 3 each), original method validation

		50% Spike		100% Spike	
Sample	Description	Mean recovery, %	RSD, %	Mean recovery, %	RSD, %
1	Milk-based infant formula powder	101.9	3.8	103.6	3.5
2	Milk-based partially hydrolyzed infant formula powder	93.7	4.4	94.6	0.2
3	Milk-based adult nutritional powder (bLF ≤14 mg/100 g)	97.1	5.6	90.3	3.0
4	Milk-based infant formula powder (bLF ≤14 mg/100 g)	92.9	6.0	92.7	3.7

		Acceptance criteria: 90–110%			

**Table 11.** bLF recovery analysis summary (*n* = 3 each), updated method validation

		50% Spike		100% Spike	
Sample	Description	Mean recovery, %	RSD, %	Mean recovery, %	RSD, %
5	Milk-based infant formula powder (bLF ≤14 mg/100 g)	97.7	4.6	93.4	3.5
6	Milk-based toddler formula powder (bLF ≤14 mg/100 g)	92.1	3.9	93.3	2.3
7	Milk-based adult nutritional powder (bLF ≤14 mg/100 g)	97.6	6.2	97.2	4.5
8	Milk-based infant formula powder, overspiked based on 193 mg/100 g bLF	94.8	1.4	96.1	0.3

		Acceptance criteria: 90–110%			

### Analytical Range

The analytical range of the lactoferrin method as determined from the range of the standard curve is 2–278 mg/100g. The sample concentrations assessed in this lactoferrin validation study ranged from 4–193 mg/100g, based on the repeatability, intermediate precision, and accuracy results.

## Conclusions

The described method has met the performance requirements defined in SMPR 2020.005 and is suitable for the quantification of soluble, intact, non-denatured bLF in bovine milk-based infant formulas, and pediatric and adult nutritional products.
